# Chondral Differentiation of Induced Pluripotent Stem Cells Without Progression Into the Endochondral Pathway

**DOI:** 10.3389/fcell.2019.00270

**Published:** 2019-11-01

**Authors:** Solvig Diederichs, Felicia A. M. Klampfleuthner, Babak Moradi, Wiltrud Richter

**Affiliations:** ^1^Research Center for Experimental Orthopaedics, Center for Orthopaedics, Trauma Surgery and Paraplegiology, Heidelberg University Hospital, Heidelberg, Germany; ^2^Clinic for Orthopaedics and Trauma Surgery, Center for Orthopaedics, Trauma Surgery and Paraplegiology, Heidelberg University Hospital, Heidelberg, Germany

**Keywords:** induced pluripotent stem cells, chondrogenesis, cartilage, hypertrophy, endochondral bone formation, Indian hedgehog

## Abstract

A major problem with chondrocytes derived *in vitro* from stem cells is undesired hypertrophic degeneration, to which articular chondrocytes (ACs) are resistant. As progenitors of all adult tissues, induced pluripotent stem cells (iPSCs) are in theory able to form stable articular cartilage. *In vitro* differentiation of iPSCs into chondrocytes with an AC-phenotype and resistance to hypertrophy has not been demonstrated so far. Here, we present a novel protocol that succeeded in deriving chondrocytes from human iPSCs without using pro-hypertrophic bone-morphogenetic-proteins. IPSC-chondrocytes had a high cartilage formation capacity and deposited two-fold more proteoglycans per cell than adult ACs. Importantly, cartilage engineered from iPSC-chondrocytes had similar marginal expression of hypertrophic markers (*COL10A1*, *PTH1R*, *IBSP*, *ALPL* mRNAs) like cartilage from ACs. Collagen X was barely detectable in iPSC-cartilage and 30-fold lower than in hypertrophic cartilage derived from mesenchymal stromal cells (MSCs). Moreover, alkaline phosphatase (ALP) activity remained at basal AC-like levels throughout iPSC chondrogenesis, in contrast to a well-known significant upregulation in hypertrophic MSCs. In line, iPSC-cartilage subjected to mineralizing conditions *in vitro* showed barely any mineralization, while MSC-derived hypertrophic cartilage mineralized strongly. Low expression of *Indian hedgehog* (*IHH*) like in ACs but rising *BMP7* expression like in MSCs suggested that phenotype stability was linked to the hedgehog rather than the bone morphogenetic protein (BMP) pathway. Taken together, unlimited amounts of AC-like chondrocytes with a high proteoglycan production reminiscent of juvenile chondrocytes and resistance to hypertrophy and mineralization can now be produced from human iPSCs *in vitro*. This opens new strategies for cartilage regeneration, disease modeling and pharmacological studies.

## Introduction

Articular cartilage has a low regenerative capacity. Focal defects frequently fail to repair and pose a severe risk to develop osteoarthritis (Buckwalter and Mankin, 1997). Cell and tissue engineering therapies were developed and optimized to meet this clinical need. Currently, these therapies depend on autologous articular chondrocytes (ACs), which are, however, highly invasive to harvest and extremely limited in supply. For pharmacological studies and disease models human ACs are often inapplicable because of their restricted access and low expandability, also impeding clonal expansion after genetic modification.

Stem cells, which are available in high numbers from less or non-invasive sources and that have the ability to form cartilage, are in the focus of the intense search for potential alternative cell sources for cartilage regeneration. Bone marrow-derived multipotent progenitor cells (frequently designated as mesenchymal stromal or stem cells, MSCs) are the best-characterized stem cell source for cartilage regeneration. A standard protocol to differentiate MSCs into chondrocytes *in vitro* has long been established (Mackay et al., 1998; Barry et al., 2001). However, instead of forming articular cartilage, MSC-chondrocytes mimic growth plate chondrocytes undergoing endochondral ossification, become hypertrophic and develop a mineralization activity. Upon implantation and vascularization at ectopic sites, this hypertrophic cartilage is transformed into bone (Pelttari et al., 2006). In contrast, when ACs are induced to cartilage formation *in vitro* under the same conditions (re-differentiation culture), they form phenotypically stable cartilage that maintains low expression of hypertrophic markers and does not form ectopic bone *in vivo*. In line with developmental mouse studies (Amizuka et al., 1994; Vortkamp et al., 1996), suppression of pro-hypertrophic Indian hedgehog (IHH) signaling by parathyroid hormone related protein was proposed as mechanism how ACs maintain phenotype stability *in vitro* (Fischer et al., 2010). In contrast, MSCs upregulate IHH-activity along with bone morphogenetic proteins (BMPs), all of which is supported by WNT-signaling (Fischer et al., 2010; Dexheimer et al., 2016; Diederichs et al., 2019). Despite intense efforts, the undesired hypertrophic degeneration of MSC-chondrocytes can currently not be prevented, and reproducible articular cartilage neogenesis from MSCs *in vitro* still remains elusive.

Pluripotent stem cells are developmental progenitors of all adult tissues. Therefore, in theory, they must be intrinsically capable to form both, phenotypically stable articular cartilage and hypertrophic mineralizing cartilage. Induced pluripotent stem cells (iPSCs) can be reprogramed from any nucleated cell and expanded in culture without losing differentiation capacity (Yamashita et al., 2018). Thus, unlimited cell quantities are available from minimally or non-invasive sources, which makes iPSCs highly attractive for cartilage regeneration. Being extremely immature, *in vitro* differentiation of iPSCs into chondrocytes is, however, highly complex and despite a large number of reports, no standard protocol has yet emerged (Tsumaki et al., 2015; Castro-Vinuelas et al., 2018). Common strategy in most protocols is a pre-differentiation into the mesodermal lineage that generates mesenchymal progenitors. These are subsequently submitted to 3D chondrogenic pellet culture adopting the protocol for MSC-chondrogenesis and AC re-differentiation (Umeda et al., 2012; Wu et al., 2013; Craft et al., 2015; Yamashita et al., 2015; Adkar et al., 2019). One major shortcoming of this approach is a considerable heterogeneity of differentiation outcome (Diederichs et al., 2016).

BMPs (e.g., BMP2, BMP4), often in combination with transforming growth factor TGF-β, are frequently used for chondrogenesis of iPSC-derived cells because of their known pro-chondrogenic activity and high relevance during embryonic cartilage formation (Umeda et al., 2012; Wu et al., 2013; Cheng et al., 2014; Yamashita et al., 2015; Nam et al., 2017; Adkar et al., 2019). In line, we previously observed that BMP4 and BMP6 enhanced human iPSC-chondrogenesis driven by TGF-β and appeared to increase SOX9 protein levels (Diederichs et al., 2016). However, BMPs did not overcome heterogeneity. Most importantly, undesired hypertrophy as indicated by collagen-X deposition and alkaline phosphatase (ALP) activity was induced. In line, studies using BMP4 as pre-chondrogenic stimulator prior to TGF-β-induced chondrogenesis or continuously applying BMP4 or BMP7 during chondrogenesis in absence of TGF-β reported *COL10A1* upregulation or endochondral stimulation for iPSC-derived cells (Wu et al., 2013; Craft et al., 2015; Adkar et al., 2019). Altogether, BMP-treatment appears inadvisable for iPSC-chondrogenesis due to its strong pro-hypertrophic activity when aiming at stable articular cartilage formation resistant to hypertrophic degeneration and mineralization.

Although studies using only TGF-β for driving chondrogenesis of iPSC-derived cells have been published, none has so far reached convincing chondrogenesis with strong matrix deposition. Either the upregulation of *COL2A1*-mRNA remained far below levels seen during standard AC-re-differentiation and MSC-chondrogenesis *in vitro* (Borestrom et al., 2014; Ko et al., 2014), or deposition of cartilage matrix with the main components collagen-II and proteoglycans remained low (Lee et al., 2015; Nejadnik et al., 2015; Suchorska et al., 2017). Since hypertrophy develops in tight conjunction with the increasing chondrogenic differentiation in MSCs (Gabler et al., 2015; Dexheimer et al., 2016), robust chondrogenesis is an essential prerequisite for addressing the potential progression into the endochondral lineage. Therefore, the question whether cartilage induced *in vitro* from iPSCs with TGF-β but without BMP-treatment will adopt an AC-like phenotype or alternatively progress into hypertrophy and endochondral ossification like MSCs, is currently completely open.

We hypothesized that TGF-β when not preceded by or combined with BMP may be able to direct iPSC-chondrogenesis into phenotypically stable non-hypertrophic articular cartilage, when the generated mesenchymal progenitors are sufficiently pre-chondrogenic. To overcome heterogenic differentiation outcomes, we here, enriched condensing cells after initiating chondrogenesis and in addition selected iPSC-chondrocytes with a strong early upregulation of *SOX9* expression. Aim was to obtain cells with a chondral phenotype where all relevant hypertrophic markers including *IHH*, collagen-X, *PTH1R*, ALP and integrin-binding sialoprotein *IBSP* are absent or as low as in re-differentiated ACs and that are resistant to mineralization. Stable articular cartilage neogenesis from iPSCs *in vitro* would provide the first functionally equivalent and unlimitedly accessible cell source as alternative for ACs. These would be ideal for numerous applications ranging from cartilage tissue engineering to functional pharmacological studies, disease modeling, and cell-based clinical therapies.

## Materials and Methods

### Cell Culture

The commercial iPS(IMR90)-4 line (WiCell) was originally reprogramed from IMR90 human fetal fibroblasts, which were extensively characterized by the ENCODE consortium (Yu et al., 2007). The D1-iPSC line which was reprogramed from healthy human fibroblasts (Larribere et al., 2015) was a kind gift from J. Utikal. IPSCs were cultured on hESC-qualified Matrigel^®^ (Corning Life Sciences) with mTeSR^TM^-1 medium (STEMCELL Technologies) as described before (Diederichs et al., 2016).

Articular chondrocytes were isolated from human articular cartilage obtained with informed consent from patients undergoing total knee replacement as described (Benz et al., 2002). The study was approved by the ethics committee on human experimentation of the Medical Faculty of Heidelberg University and was in agreement with the Helsinki Declaration of 1975 in its latest version. Chondrocytes were expanded in low-glucose DMEM (Gibco^TM^, Life Technologies) supplemented with 10% FCS (Gibco^TM^) and 100 U/mL penicillin, 100 μg/mL streptomycin (Biochrom) until passage 2.

Mesenchymal stromal cells were isolated from fresh human bone marrow aspirates obtained with informed consent from patients undergoing total hip replacement surgery as described (Winter et al., 2003). MSCs were expanded in high-glucose DMEM, 12.5% FCS, penicillin/streptomycin, 2mM L-glutamine (Gibco^TM^), 1% non-essential amino acids (Gibco^TM^), 1% β-mercaptoethanol (Gibco^TM^), and 4 ng/mL human FGF-2 (Miltenyi Biotec) until passage 3. MSCs were routinely stained for surface markers CD73, CD90, CD105 and the absence of CD34 and CD45 (data not shown).

Induced pluripotent stem cells were induced into mesenchymal progenitors as described before (Diederichs et al., 2016) by treatment and subculture in MSC expansion medium until passage 3. 10 μM ROCK-inhibitor Y27632 (STEMCELL Technologies) were used for 24–48 h when seeding into passages 0–2. At this stage, expression of pluripotency-associated genes was below detection limit or similar to levels in MSCs according to data from a whole-genome microarray (Supplementary Figure S1A; Buchert et al., 2019). Like MSCs, iPSC-derived progenitors expressed the characteristic MSC surface markers *CD44*, *CD73*, *CD105* (Supplementary Figure S1A; Diederichs and Tuan, 2014; Diederichs et al., 2016).

For chondrogenesis, 5 × 10^5^ mesenchymal progenitors were pelleted in chondrogenic medium consisting of high-glucose DMEM, 0.1 mM dexamethasone, 0.17 mM ascorbic acid 2-phosphate, 1mM sodium pyruvate, 0.35 mM proline, 1.25 mg/mL BSA (all from Sigma-Aldrich), penicillin/streptomycin, 5 mg/mL transferrin, 5 ng/mL sodium selenite, 5 mg/mL insulin (Lantus^®^, Sanofi-Aventis) or ITS^TM^ + premix (Corning), and 10 ng/mL TGF-β1 (Miltenyi). Lose cells that did not contribute to the condensing pellet were flushed away during medium exchanges, thus enriching for condensing cells. Experiments were only included, when *SOX9* expression at day 14 of chondrogenesis was upregulated over the threshold of 20% mean reference expression (*HPRT*, *CPSF6*, *RPL13*). Preliminary tests had shown that this *SOX9* threshold correlated with strong induction of *COL2A1* (300% relative expression at day 14), and a good outcome of chondrogenesis at day 42 according to cartilage-specific histology. Twelve out of 21 mesenchymal progenitor lines derived from IMR-iPSCs and only 1 out of 6 D1-iPSC-derived lines passed this quality test. AC re-differentiation and MSC-chondrogenesis were performed under similar conditions.

For *in vitro* mineralization, 6-week-pellets were transferred into mineralizing medium supplemented with 1 nM L-thyroxine (Sigma-Aldrich) and 10 mM β-glycerophosphate (HM in Kunisch et al., 2018) for additional 8 weeks.

### Gene Expression Analysis

RNA was extracted via standard guanidinium thiocyanate/phenol/chloroform protocol using TriFast (Peqlab) and reverse-transcribed using OmniScript^®^ (Qiagen). Transcript levels were determined by quantitative PCR using Light Cycler^TM^ technology (Roche Diagnostics) with the primers given in Supplementary Table S1. Relative expression was calculated as 100%⋅1.8^ΔC_t_^ with *CPSF6* as reference gene.

### Proteoglycan Quantification

Pellets were digested with proteinase-K and proteoglycans stained with 1,9-dimethyl-methylene-blue and referred to DNA measured via Quant-iT PicoGreen-dsDNA-Assay as described before (Diederichs et al., 2019).

### Histology

Formaldehyde-fixed pellets were dehydrated and paraffin-embedded. Five-micrometer sections were stained with safranin-O (0.2% in 1% acetic acid) or alizarin red-S (0.5% in water) with fast green counter-staining (0.04% in 0.2% acetic acid). Collagen immunohistochemistry was performed as described (Diederichs et al., 2019) using anti-collagen-type-II (clone 4c11, MP Biomedicals/Quartett, 1:1000), and anti-collagen-type-X (clone X-53, Quartett, 1:10) and biotinylated goat anti-mouse antibody (Dianova) followed by streptavidin-ALP and fast red detection (Roche). Nuclei were counterstained with hematoxylin. Aggrecan was stained with anti-human aggrecan antibody (HAG7D4; 1:25; Acris in PBS/1% BSA) as described (Hesse et al., 2018). ALP activity was stained via conversion of the ALP substrates NBT and BCIP (2% in phosphate saline buffer; Roche).

### Collagen Isolation and Western Blotting

Collagens were salted out, precipitated and re-solubilized from pepsin-digested pellets as described before (Fischer et al., 2010). This degraded most proteins including common references like actin and GAPDH. Collagens were separated via SDS-PAGE and blotted onto a nitrocellulose membrane (GE Healthcare) and detected with the same antibodies used in histology. Bands were visualized with peroxidase-coupled secondary antibodies using ECL detection.

### ALP Activity in Culture Supernatants

Culture supernatants were pooled from 3 to 6 pellets per group and ALP activity measured via conversion of p-nitrophenylphosphate as described (Diederichs et al., 2019).

### Microcomputed Tomography

Pellets were scanned against air on styrofoam with a SkyScan-1076 microtomograph using no filter (voxel size 8.85 μm, 40kV, 250 μA, 900ms, frame averaging 3, 360° scan). Reconstruction was performed with NRecon^®^ (version 1.6.3.2, Skyscan). Total volume (gray scale 25–255) and mineralized volume (70–255) were calculated with CTAn^®^. These settings detected a mineralization volume of 0 at day 0 of *in vitro* mineralization.

### Statistics

The number of independent iPSC-derived cell lines as well as the number of independent donor populations of MSCs and ACs used for each test is given in the figure captions. Mean and standard error of the mean (SEM) were calculated. Regulation over time within one group and differences between more than two groups were assessed with one-way ANOVA and *post hoc* LSD correction. Comparisons between two groups at the same time point were performed with Student’s *t*-test.

## Results

**FIGURE 1:**
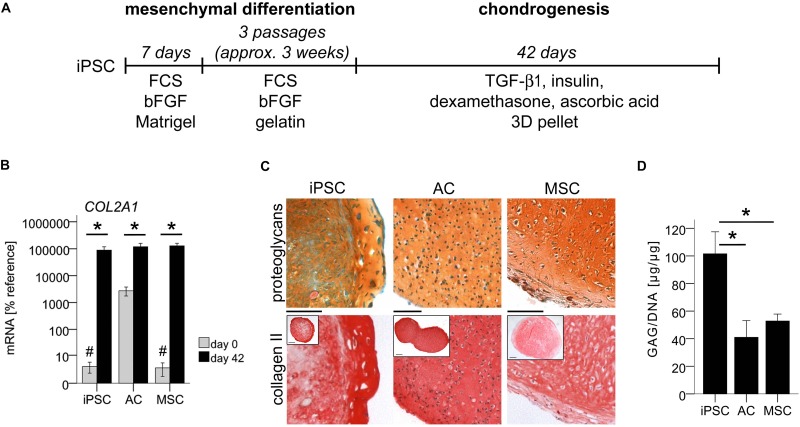
*In vitro* generation of cartilage from iPSCs. Subsequent to mesenchymal differentiation, 500,000 iPSC-derived cells or expanded ACs or MSCs were treated in 3D pellet culture with TGF-β1 in chondrogenic medium. **(A)** Schematic overview of iPSC differentiation. **(B)** Expression of *COL2A1* relative to the reference gene *CPSF6* assessed by qPCR at the beginning (day 0) and end (day 42) of chondrogenesis. Data are presented as mean ± SEM of *n* = 7 independent IMR-iPSC-derived cell lines and *n* = 5 AC or MSC donor populations. *p* < 0.05 compared to day 0 (^∗^), or to ACs at day 0 (#) according to LSD-corrected ANOVA. **(C)** Histological assessment of proteoglycans with safranin O staining and collagen II content with anti-human collagen II immune histology. Representative pictures of *n* = 6 independent IMR-iPSC-derived lines and *n* = 5 AC or MSC donor populations. Scale bars represent 100 and 200 μm in overviews. **(D)** Quantification of the proteoglycan amount according to DMMB assay relative to total DNA amount according to PicoGreen assay. Data are given as mean ± SEM of *n* = 4 independent IMR-iPSC-derived lines, *n* = 4 AC donor populations, and *n* = 5 MSC donor populations measured in biological duplicates. ^∗^*p* < 0.05 according to LSD-corrected ANOVA.

Human iPSCs were differentiated into mesenchymal progenitors in the presence of serum and 4 ng/mL FGF-2, and subsequently subjected to pellet culture with 10 ng/mL TGF-β1 in serum-free chondrogenic medium (Figure 1A). Lose cells not contributing to pellet-aggregation were flushed away to enrich condensing cells. To overcome heterogenic differentiation outcomes, experiments were only continued beyond 2 weeks when *SOX9*-mRNA was upregulated over the threshold level of 20% mean reference gene expression. After 6 weeks of chondrogenesis, iPSC-derived cells had significantly upregulated *COL2A1*-expression by over 24,000-fold (Figure 1B). In comparison, ACs started into re-differentiation with 860-fold higher *COL2A1*-expression and upregulation was 90-fold, so that the iPSC group caught up over time, reaching ACs by day 42. Such levels were also obtained for MSCs used as control for stem cell chondrogenesis (Figure 1B). Also, *ACAN*-expression was significantly upregulated in iPSC-derived cells and reached levels of re-differentiated ACs by day 42 (Supplementary Figure S1B).

Induced pluripotent stem cell-pellets were smaller than AC- and MSC-pellets (Figure 1C, insets) in line with previous data (Buchert et al., 2019). Histology revealed strong deposition of cartilaginous matrix rich in proteoglycans, collagen-II, and aggrecan similar to AC- and MSC-derived cartilage (Figure 1C and Supplementary Figure S1C). Often, iPSC-constructs showed a morphologically distinct outer ring comprised of multiple cell layers with strong cartilaginous matrix. DMMB assay demonstrated a mean proteoglycan content of 100 μg/μg DNA in iPSC-constructs, which was two-fold higher than in AC- or MSC-cartilage (Figure 1D). Overall, this documented a superior proteoglycan production at similar *COL2A1*-expression in iPSC-chondrocytes demonstrating that they can outperform the cartilage formation capacity of ACs and MSCs. This strong cartilage formation ruled out that low hypertrophy may be disguised by insufficient chondrogenesis of cells. Therefore, we next investigated whether iPSC-cartilage adopted a non-hypertrophic articular phenotype or progressed into undesired endochondral ossification similar to MSCs.

### Non-hypertrophic, ALP-Free Chondrocytes From iPSCs

**FIGURE 2:**
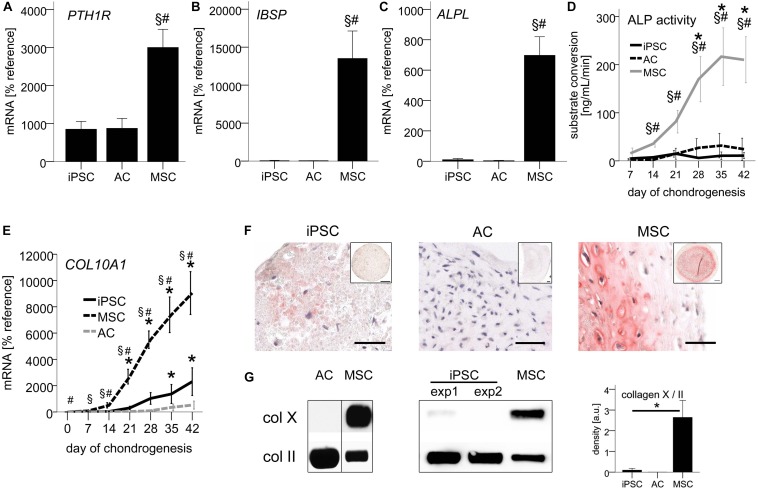
Non-hypertrophic phenotype of iPSC-chondrocytes similar to re-differentiated ACs. 500,000 cells were treated with TGF-β1 in chondrogenic medium in 3D pellet culture. **(A–C)** QPCR signals relative to mean expression of the reference gene *CPSF6*. Data are presented as mean ± SEM of *n* = 5 AC and MSC donor populations and *n* = 3–5 independent IMR-iPSC-derived lines. **(D)** ALP activity in culture supernatants measured via conversion of the substrate p-nitrophenyl phosphate. Data are presented as mean ± SEM of *n* = 7 independent IMR-iPSC-derived lines and *n* = 4 MSC and AC donor populations. ^∗^*p* < 0.05 compared to day 0, §*p* < 0.05 compared to the AC group, #*p* < 0.05 compared to the iPSC group according to LSD-corrected ANOVA. **(E)** QPCR signals of *COL10A1* relative to mean expression of the reference gene *CPSF6*. Data are presented as mean ± SEM of *n* = 3–7 independent IMR-iPSC-derived lines and *n* = 5 MSC and AC donor populations. *p* < 0.05 compared to day 0 (^∗^), compared to the iPSC group (#), or compared to the AC group at the same time point (§) according to LSD-corrected ANOVA. **(F)** Immune histological assessment of collagen-X. Representative pictures of *n* = 9 independent IMR-iPSC-derived lines and *n* = 6 MSC populations. Scale bars represent 50 μm in magnifications and 200 μm in overviews. **(G)** Collagen-X levels according to Western blot detection using collagen II as reference. Collagens extracted after pepsin digestion from iPSC or MSC pellets at day 42 were loaded. The membrane was cut at 75 kDa and the two parts stained separately for collagen-X and II. Western blot band density was semiquantified; bars represent mean ± SEM of *n* = 7 independent IMR-iPSC-derived lines and *n* = 2 MSC donors measured in duplicates or triplicates. ^∗^*p* < 0.05 according to LSD-corrected ANOVA. Original gels provided in Supplementary Figure S3.

First, we assessed whether markers typically associated with MSC-hypertrophy are expressed in iPSC-chondrocytes. PCR revealed that in the iPSC group *PTH1R*, *IBSP*, and *ALPL* were expressed as low as in re-differentiated ACs, while significantly higher expression was observed in the MSC group (*PTH1R*: 4-fold, *IBSP*: 275-fold, *ALPL*: 70-fold; Figures 2A–C). In line with low *ALPL* gene expression, ALP activity, which is highly relevant for tissue mineralization, remained at basal levels throughout iPSC chondrogenesis similar to AC re-differentiation (Figure 2D). As expected, a significant upregulation of ALP activity was observed during MSC chondrogenesis, reaching almost 20-fold higher levels than the iPSC group at day 42. IPSC-chondrocytes maintained these low ALP levels even during prolonged chondrogenic culture for up to 8 weeks (data not shown). Absence of ALP activity in iPSC-chondrocytes was also confirmed in a second independent iPSC-line, D1-iPSCs (Supplementary Figure S2). Altogether, these data demonstrated that iPSC-derived chondrocytes underwent a chondral and not endochondral differentiation and adopted a phenotype similar to re-differentiated ACs.

### AC-Like COL10A1 Expression and Low Collagen-X Deposition

Focusing next on *COL10A1* expression via qPCR, we observed only little upregulation of mRNA levels by day 42, both during iPSC-chondrogenesis and *in vitro* AC-re-differentiation with no significant differences between groups (*p* = 0.714 at day 42, Figure 2E). In contrast, during MSC chondrogenesis, *COL10A1* was significantly upregulated over time and was at all time points significantly higher than during iPSC-chondrogenesis and AC re-differentiation.

Histology showed a very faint staining for collagen-X protein in IMR-iPSC-cartilage, while D1-iPSC cartilage and AC-cartilage remained negative (Figure 2F and Supplementary Figure S2). In contrast, strong staining was observed in MSC-cartilage. For quantitative analysis, we extracted collagens from differentiated pellets and loaded samples standardized for collagen-II levels in Western blot analysis, detecting collagen-X levels in the same sample on the same membrane. As expected, AC-cartilage was negative for collagen-X, while a strong band was observed for hypertrophic MSC-pellets (Figure 2G). For iPSC-cartilage, in 5/7 independent IMR-iPSC-derived lines no collagen-X was observed, while a faint band was observed in 2/7 lines. Semiquantification of Western blots revealed no significant difference between iPSC and AC-cartilage, while a 30-fold higher collagen-X/II ratio was obtained in MSC-pellets (Figure 2G). This again demonstrated that iPSC-chondrocytes closely mimicked re-differentiated ACs and showed an overall non-hypertrophic phenotype.

### Resistance of iPSC-Chondrocytes to Mineralization

**FIGURE 3:**
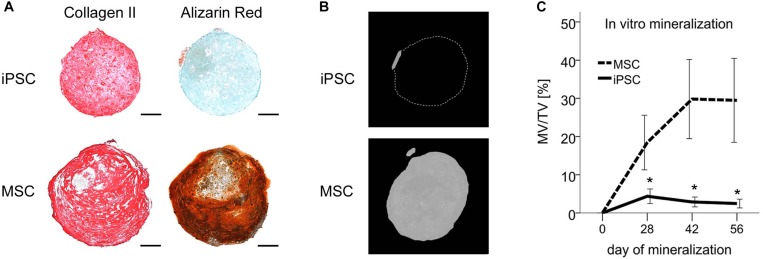
Resistance of iPSC-derived cartilage but not MSC-derived hypertrophic cartilage to mineralization *in vitro*. After 42 days of chondrogenesis pellets derived from IMR-iPSCs or MSCs were transferred to mineralizing medium for additional 8 weeks. **(A)** Immune histology for collagen-II and detection of mineralization with alizarin red after 8 weeks of mineralization. Scale bars represent 200 μm. **(B)** Micro-CT reconstructions after 8 weeks of mineralization. Scale bars represent 200 μm. **(C)** Mineralized volume per total volume during *in vitro* mineralization. Data are given as mean ± SEM. *N* = 7 independent IMR-iPSC–derived lines assessed in biological duplicates or triplicates and *n* = 4 MSC donor populations assessed in biological duplicates. ^∗^*p* < 0.05 compared to MSCs at the same time point according to *t*-test.

To verify that the non-hypertrophic phenotype of iPSC-chondrocytes came with a resistance to mineralization, iPSC-derived pellets were transferred after 6 weeks of chondrogenesis to mineralizing medium containing L-thyroxine and β-glycerophosphate. After 8 weeks, mineralization remained undetectable in 14/18 pellets assessed by histology (Figure 3A). In line, the mineralized volume remained below 3% of the total volume according to micro-CT in 18/20 pellets (Figure 3B). In contrast, strong mineralization was detected in pellets from all four independent MSC donor populations via histology as expected, reaching up to 70% (mean 29% ± 31%) of the total volume according to micro-CT. The mineralized volume per total volume was significantly lower in iPSC-cartilage than in MSC-cartilage at all tested time points (12-fold at day 56, *p* < 0.05, Figure 3C). Together, this demonstrated an overall resistance to mineralization of iPSC-cartilage while MSC-derived hypertrophic cartilage showed a strong mineralizing activity.

### AC-Like IHH Expression in Non-hypertrophic iPSC-Chondrocytes

**FIGURE 4:**
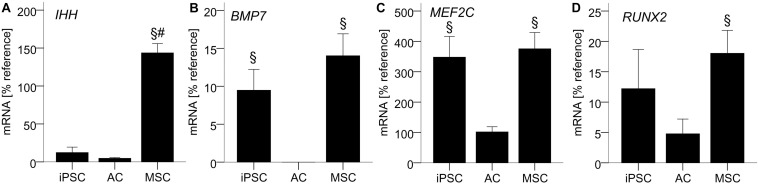
AC-like *IHH* expression in iPSC-derived chondrocytes. 500,000 cells were treated with TGF-β1 in chondrogenic medium in 3D pellet culture. QPCR signals for *IHH* **(A)**, *BMP7* **(B)**, *MEF2C* **(C)**, and *RUNX2* **(D)** relative to mean expression of the reference gene *CPSF6*. Data are presented as mean ± SEM of *n* = 5 independent AC and MSC donor populations and *n* = 3–5 independent IMR-iPSC-derived lines. §*p* < 0.05 compared to the AC group, #*p* < 0.05 compared to the iPSC group according to LSD-corrected ANOVA.

To illuminate how iPSC-chondrocytes may maintain their non-hypertrophic phenotype, we next assessed growth and transcription factors that were previously discussed to drive MSCs into hypertrophy. Importantly, *IHH-*mRNA in iPSC-chondrocytes was as low as in re-differentiated ACs, while MSC-chondrocytes expressed 15-fold higher levels (Figure 4A). In contrast, *BMP7* and *MEF2C-*mRNA levels were similar in iPSC- and MSC-chondrocytes and significantly higher than in re-differentiated ACs (Figures 4B,C). *RUNX2* was significantly higher in differentiated MSCs than in ACs, while iPSC-chondrocytes expressed intermediate levels that were not significantly different from ACs or MSCs (Figure 4D). Together, this suggested that the non-hypertrophic differentiation of iPSC-chondrocytes was linked to the absence of *IHH* expression rather than low *BMP7*, *MEF2C* or *RUNX2*-mRNA levels.

## Discussion

The attractiveness of iPSCs for cartilage regeneration roots in their abundant availability combined with their theoretic ability to form articular cartilage. Yet to date, this theory lacks practical verification, and resistance of iPSC-cartilage to undesired endochondral development remained largely unaddressed. To the best of our knowledge, this is the first report demonstrating the generation of non-hypertrophic AC-like chondrocytes with a high cartilage-forming activity from human iPSCs. This was achieved by a rather simple protocol compared to other approaches. An initial mesodermal differentiation of iPSCs in serum and FGF-2-containing monolayer culture on matrigel/gelatin was followed by TGF-β-mediated chondrogenesis in 3D-pellets. Having observed hypertrophy in iPSC-chondrocytes treated with BMPs in a previous study (Diederichs et al., 2016), we here, explicitly resigned from using BMPs before and during chondrogenesis and instead used TGF-β as only growth factor to drive chondrogenesis. IPSC-chondrocytes demonstrated an extremely high proteoglycan production per cell which outperformed adult re-differentiated ACs and MSC-chondrocytes. This reminded of juvenile chondrocytes which are known to be anabolically more active than adult chondrocytes (Aigner et al., 2007) and was in line with previous data indicating that iPSC-derivatives remained rejuvenated throughout differentiation (Frobel et al., 2014; Chan et al., 2018). Most importantly, iPSC-chondrocytes underwent a chondral not an endochondral differentiation and adopted an AC-like non-hypertrophic phenotype, as demonstrated by no or little expression of hypertrophic markers similar to ACs. This combination of high cartilage formation and resistance to endochondral differentiation is a major step toward application of iPSC-chondrocytes for cartilage repair, pharmacological studies and disease modeling.

IPSC-derived mesenchymal progenitors are known to fulfill typical requirements for MSCs (Villa-Diaz et al., 2012; Diederichs and Tuan, 2014; Hynes et al., 2014; Buchert et al., 2019) and are, for this reason, often designated as “iMSCs” (Spitzhorn et al., 2019). We and others previously reported that, despite such apparent similarities, mesenchymal progenitors derived from iPSCs are still functionally different from MSCs (Diederichs and Tuan, 2014; Frobel et al., 2014; Diederichs et al., 2016; Buchert et al., 2019). Specifically, we previously proposed that a shortage of extracellular matrix and integrin ligand expression together with insufficient pro-survival ERK1/2 activity in iPSC-derived mesenchymal progenitors causes an increased cell loss during early chondrogenesis and thus a much smaller pellet size compared to MSCs (Buchert et al., 2019). Because chondrogenic conditions were identical for iPSC-derived mesenchymal progenitors, MSCs and ACs in the current study, the expansion phase and resulting characteristics of the initial population at the start of chondrogenesis must have been decisive whether the chondral or the endochondral differentiation route was induced by TGF-β. Importantly, we here, demonstrated that our iPSC-derived mesenchymal progenitors were potent enough to undergo chondrogenesis in the presence of TGF-β without pro-hypertrophic BMP-application.

Previous studies attempted a more stringent guidance of iPSC-differentiation into chondrocytes by modulating signaling pathways relevant during embryonic cartilage development including WNT, activin-A/Nodal, TGF-β, FGF, PDGF, and importantly also BMP. Yet, so far, reproducibility and stringency of such protocols were still insufficient, and elaborate enrichment steps like cell sorting or manual picking were necessary (Umeda et al., 2012; Wu et al., 2013; Yamashita et al., 2015; Adkar et al., 2019). Protocol variety and limited reproducibility further indicate that our current understanding of the necessary pathway modulations is still incomplete. In comparison to such multiple pathway modulations, our protocol is far less elaborate, using FGF-2 and serum for mesenchymal differentiation in successive monolayer culture on two distinct hydrogels and TGF-β to drive chondrogenesis in 3D-pellet culture. Enrichment of condensing cells after chondrogenic induction was easily achieved without cell sorting or manual selection simply by flushing away loose cells that were not tightly attached to the aggregated pellets during medium exchanges. Reproducibility of chondrogenesis was further increased by selecting for strong upregulation of *SOX9*, which we previously identified as a shortcoming of iPSC chondrogenesis (Diederichs et al., 2016). Altogether, we believe that our resigning from BMP treatment was key to obtaining chondral instead of endochondral differentiation. Although efficiency and reproducibility of *in vitro* iPSC-chondrogenesis still need improvements also with our protocol, we here, demonstrated that this effort can be rewarded by obtaining non-hypertrophic AC-like chondrocytes.

Low expression of *IHH* made us believe that the non-hypertrophic phenotype in iPSC-chondrocytes may be linked with suppression of the pro-hypertrophic hedgehog pathway. In a previous study, we demonstrated that during MSC-hypertrophy *IHH* upregulation is partly driven by WNT-activity (Diederichs et al., 2019). WNT-activity in turn was shown to be modulated by proteoglycans (Ai et al., 2003) and we have recently described that WNT/β-catenin levels declined with increasing proteoglycan content both in AC- and MSC-cartilage (Praxenthaler et al., 2018; Diederichs et al., 2019). In addition, proteoglycans were indicated to modulate receptor-binding of TGF-β (Hildebrand et al., 1994) and also BMP2-activity (Ruppert et al., 1996), as reviewed elsewhere (van der Kraan et al., 2002; Melrose et al., 2016). Thus, the particularly high proteoglycan deposition by iPSC-chondrocytes may have contributed to their non-hypertrophic phenotype by modulating TGF-β activity and suppressing pro-hypertrophic WNT and BMP-activity. Thus, further studies to demonstrate functional causality between high proteoglycan deposition, low IHH levels and chondral development without hypertrophic degeneration of iPSC-cartilage should include investigating the TGF-β, BMP, and WNT signaling-activity.

Remaining small shortcoming was that collagen-X deposition, although indeed very low, was still not fully absent as routinely observed for ACs. Previously we demonstrated that epigenetic regulation by DNA-methylation was important for *COL10A1* silencing in ACs. While in ACs all possible DNA-methylation sites in the *COL10A1* promoter were completely methylated, in MSCs two putative binding sites for the transcription factor MYC remained demethylated (Zimmermann et al., 2008). In addition, *MYC*-overexpression in MSCs correlated with an increase of *COL10A1/COL2A1*-mRNA, strongly suggesting a functional relevance of the MYC transcription factor for *COL10A1*-expression (Melnik et al., 2019). Thus, it is tempting to speculate that lack of promoter methylation in iPSC-chondrocytes may be linked to the leaky *COL10A1*-expression. For now it must be stressed, that experience with MSC-chondrogenesis proved collagen-X very hard to modulate and apparently impossible to uncouple from desired collagen-II deposition. Long-lasting and laborious anti-hypertrophic treatments like daily PTHrP-pulses or WNT-inhibition were incapable of lowering collagen-X deposition during MSC-chondrogenesis (Fischer et al., 2016; Diederichs et al., 2019). Although BMP-inhibition with dorsomorphin could, this came at the expense of co-suppressing collagen-II (Dexheimer et al., 2016). We here, achieved strong collagen-II accompanied by only minimal collagen-X during iPSC-chondrogenesis and the enormous extent of this advance is underscored by the numerous previous failures with MSC-chondrogenesis. With some additional optimization, we hope that generation of non-hypertrophic articular cartilage free of collagen-X from iPSCs may be close to reality.

## Conclusion

This is the first experimental demonstration of a chondral *in vitro* differentiation of human iPSCs into chondrocytes resistant to hypertrophic degeneration like AC-derived cartilage. This was achieved by BMP-free generation of iPSC-derived mesenchymal progenitors with a high chondrogenic capacity, which allowed driving chondrogenesis with TGF-β without addition of pro-hypertrophic BMPs. The resulting iPSC-chondrocytes were highly reminiscent of juvenile chondrocytes and deposited more proteoglycans per cell than adult ACs. Importantly, hypertrophic markers in iPSC-cartilage were absent or as low as in AC-tissue both on expression and protein level, thus documenting the absence of undesired hypertrophic degeneration that disqualifies MSCs for clinical cartilage regeneration. Although further efforts are needed to increase efficiency and reproducibility of iPSC *in vitro* chondrogenesis, our data show that this will be highly rewarding, because iPSC-chondrocytes are the first stem cell-derived chondrocytes which show a similar resistance to hypertrophy as primary ACs. Thus, our results are a major step toward novel iPSC-based cartilage therapies, genetic disease models for numerous chondrodysplasias, pharmacological drug screening platforms and for basic research models of cartilage development.

## Data Availability Statement

The raw data supporting the conclusions of this manuscript will be made available by the authors, without undue reservation, to any qualified researcher upon request.

## Ethics Statement

The studies involving human patients were reviewed and approved by the Ethics Committee on Human Experimentation of the Medical Faculty of Heidelberg University. The patients provided their written informed consent to participate in this study.

## Author Contributions

SD: conception and design, financial support, data analysis and interpretation, manuscript writing, and final approval of the manuscript. FK: collection of the data, manuscript writing, and final approval of manuscript. BM: provision of study material and final approval of manuscript. WR: conception and design, financial support, administrative support, data interpretation, and final approval of manuscript.

## Conflict of Interest

The authors declare that the research was conducted in the absence of any commercial or financial relationships that could be construed as a potential conflict of interest.
