# Supplementary material for: Chondral Differentiation of Induced Pluripotent Stem Cells Without Progression Into the Endochondral Pathway
Source: Front Cell Dev Biol. 2019 Nov 1;7:270. doi: 10.3389/fcell.2019.00270 (PMC6838640; doi:10.3389/fcell.2019.00270)
Supplement: Supplementary file 1 [file Presentation_1.PPTX]

## Slide 1
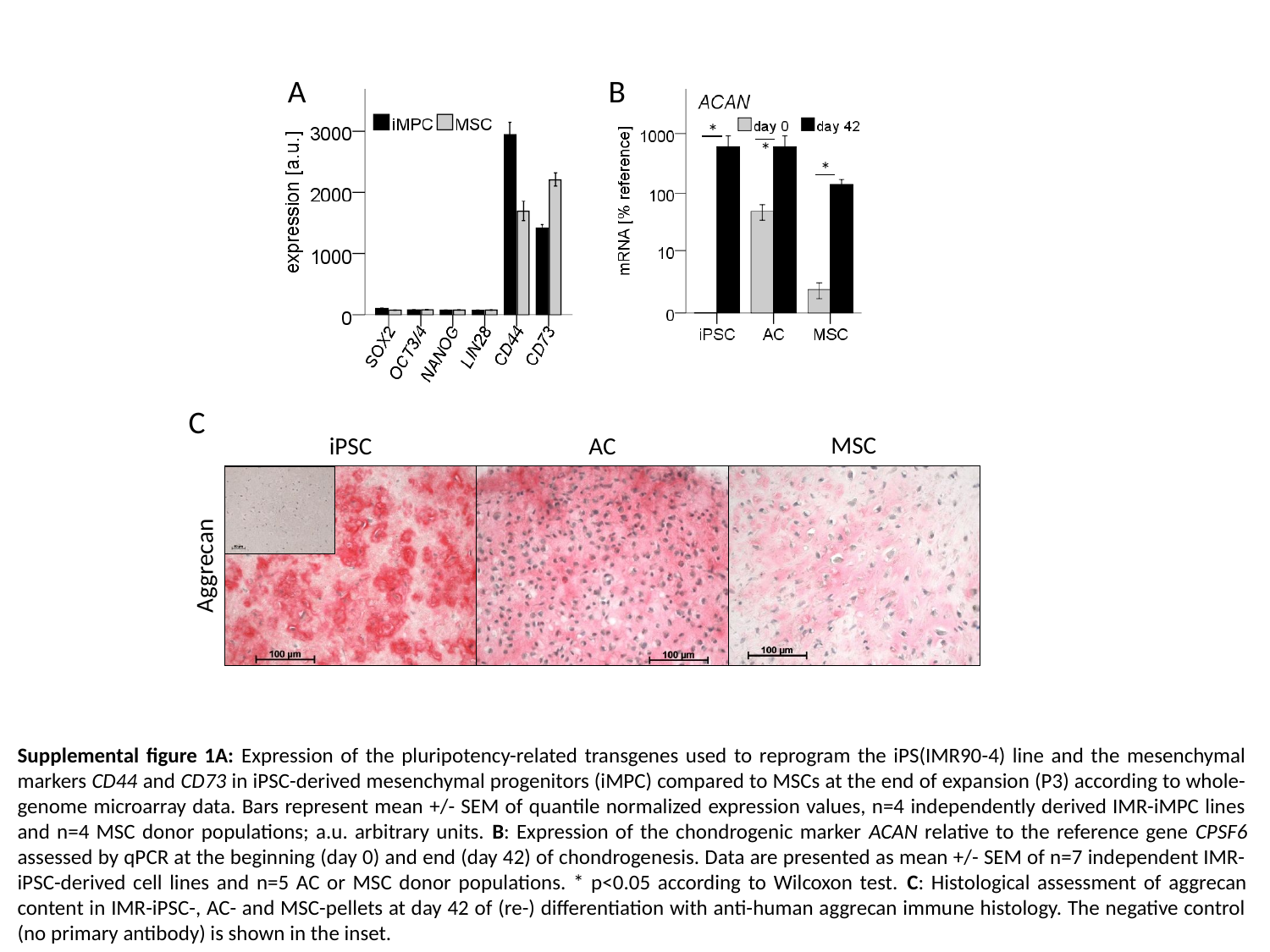

A
B
*
*
*
C
MSC
iPSC
AC
Aggrecan
Supplemental figure 1A: Expression of the pluripotency-related transgenes used to reprogram the iPS(IMR90-4) line and the mesenchymal markers CD44 and CD73 in iPSC-derived mesenchymal progenitors (iMPC) compared to MSCs at the end of expansion (P3) according to whole-genome microarray data. Bars represent mean +/- SEM of quantile normalized expression values, n=4 independently derived IMR-iMPC lines and n=4 MSC donor populations; a.u. arbitrary units. B: Expression of the chondrogenic marker ACAN relative to the reference gene CPSF6 assessed by qPCR at the beginning (day 0) and end (day 42) of chondrogenesis. Data are presented as mean +/- SEM of n=7 independent IMR-iPSC-derived cell lines and n=5 AC or MSC donor populations. * p<0.05 according to Wilcoxon test. C: Histological assessment of aggrecan content in IMR-iPSC-, AC- and MSC-pellets at day 42 of (re-) differentiation with anti-human aggrecan immune histology. The negative control (no primary antibody) is shown in the inset.

## Slide 2
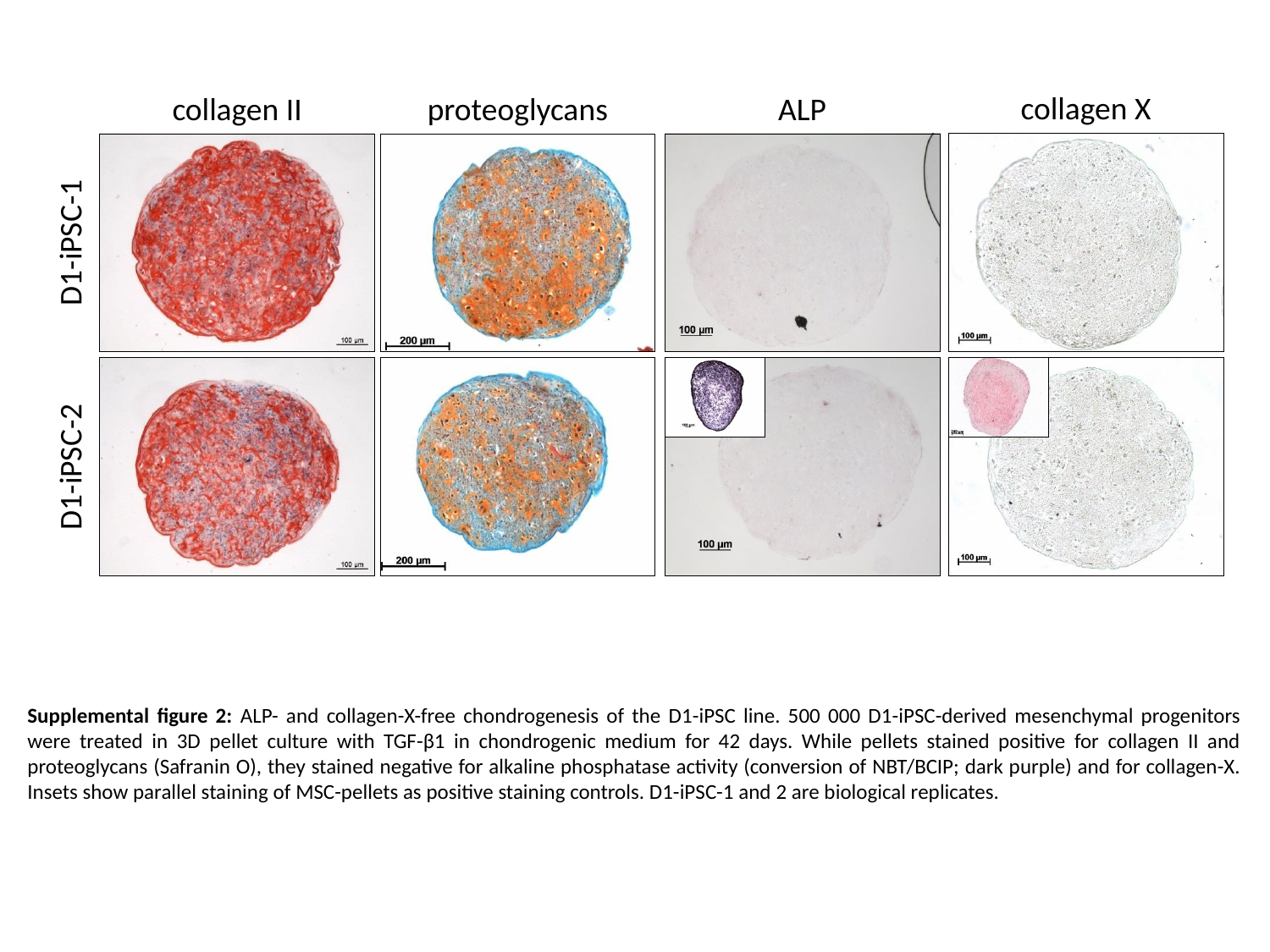

collagen X
ALP
proteoglycans
collagen II
D1-iPSC-1
D1-iPSC-2
Supplemental figure 2: ALP- and collagen-X-free chondrogenesis of the D1-iPSC line. 500 000 D1-iPSC-derived mesenchymal progenitors were treated in 3D pellet culture with TGF-β1 in chondrogenic medium for 42 days. While pellets stained positive for collagen II and proteoglycans (Safranin O), they stained negative for alkaline phosphatase activity (conversion of NBT/BCIP; dark purple) and for collagen-X. Insets show parallel staining of MSC-pellets as positive staining controls. D1-iPSC-1 and 2 are biological replicates.

## Slide 3
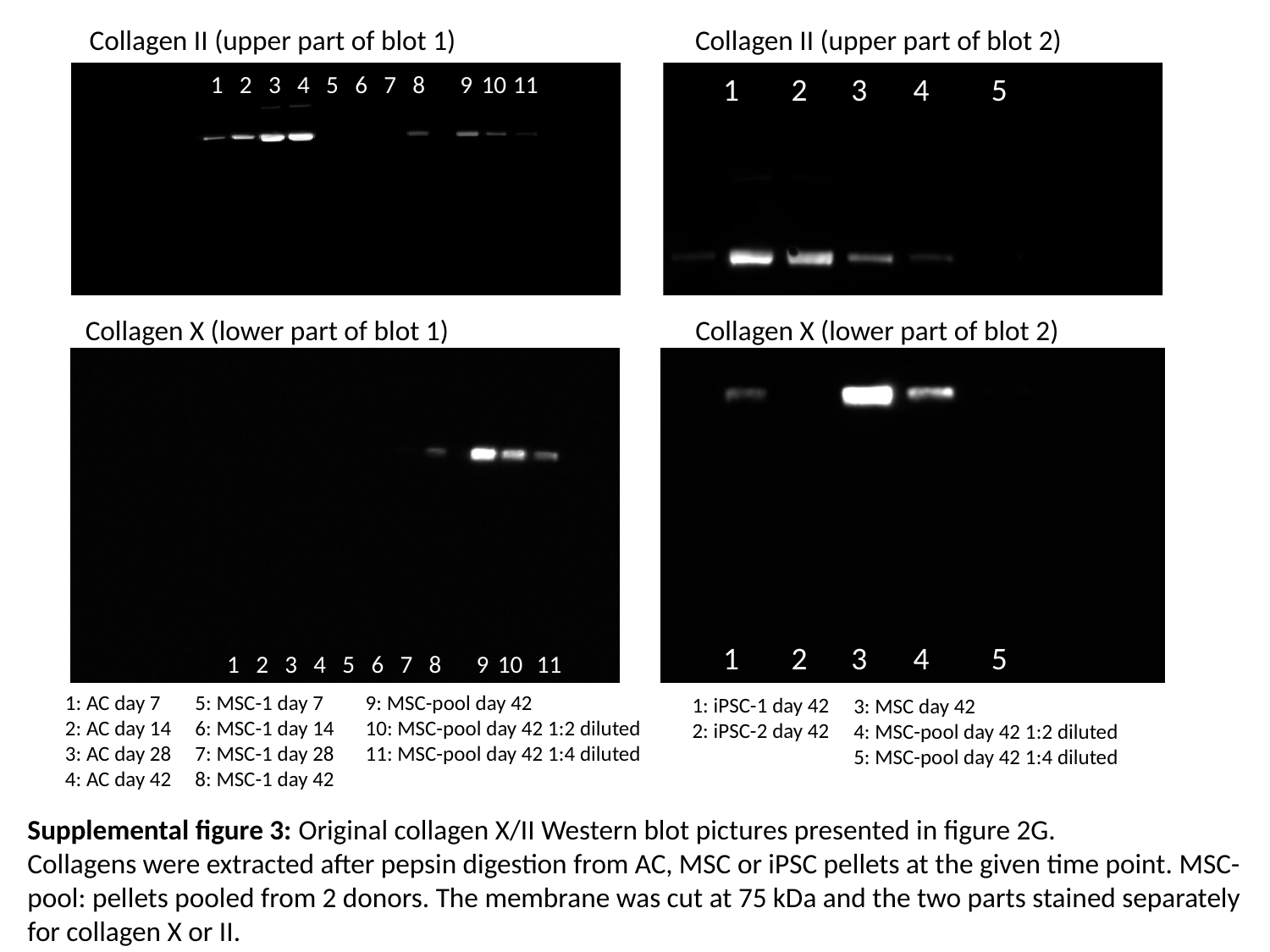

Collagen II (upper part of blot 1)
Collagen II (upper part of blot 2)
1
2
3
4
5
6
7
8
9
10
11
1
2
3
4
5
Collagen X (lower part of blot 1)
Collagen X (lower part of blot 2)
1
2
3
4
5
1
2
3
4
5
6
7
8
9
10
11
1: AC day 7
2: AC day 14
3: AC day 28
4: AC day 42
5: MSC-1 day 7
6: MSC-1 day 14
7: MSC-1 day 28
8: MSC-1 day 42
9: MSC-pool day 42
10: MSC-pool day 42 1:2 diluted
11: MSC-pool day 42 1:4 diluted
1: iPSC-1 day 42
2: iPSC-2 day 42
3: MSC day 42
4: MSC-pool day 42 1:2 diluted
5: MSC-pool day 42 1:4 diluted
Supplemental figure 3: Original collagen X/II Western blot pictures presented in figure 2G.
Collagens were extracted after pepsin digestion from AC, MSC or iPSC pellets at the given time point. MSC-pool: pellets pooled from 2 donors. The membrane was cut at 75 kDa and the two parts stained separately for collagen X or II.
