# Supplementary material for: Chondral Differentiation of Induced Pluripotent Stem Cells Without Progression Into the Endochondral Pathway
Source: Front Cell Dev Biol. 2019 Nov 1;7:270. doi: 10.3389/fcell.2019.00270 (PMC6838640; doi:10.3389/fcell.2019.00270)
Supplement: Supplementary file 2 [file Table_1.DOCX]

Supplemental Table 1: primer sequences

| Gene | fwd | rev |
| --- | --- | --- |
| *ALPL* | 5´-caccaacgtggctaagaatg-3´ | 5´-atctccagcctggtctcctc-3´ |
| *BMP7* | 5´-ccagaaccgctccaagac-3´ | 5´-gttggtggcgttcatgtag-3´ |
| *COL10A1* | 5´-tttacgctgaacgataccaaa-3´ | 5´-ttgctctcctcttactgctat-3´ |
| *COL2A1* | 5`-tggcctgagacagcatgac-3‘ | 5‘-agtgttgggagccagattgt-3‘ |
| *CPSF6* | 5‘-aagattgccttcatggaattgag-3‘ | 5‘-tcgtgatctactatggtccctctct-3‘ |
| *IBSP* | 5´-cagggcagtagtgactcatcc-3´ | 5´-tcgattcttcattgttttctcct-3´ |
| *IHH* | 5´-cgaccgcaataagtatggac-3´ | 5´-ggtgagcgggtgtgagtg-3´ |
| *MEF2C* | 5‘-gtatggcaatccccgaaact-3‘ | 5‘-atcgtattcttgctgcctgg-3‘ |
| *PTH1R* | 5´-ggtgaggtggtggctgt-3´ | 5´-agcatgaaggacaggaac-3´ |
| *RUNX2* | 5´-actctaccaccccgctgtc-3´ | 5´-cagaggtggcagtgtcatca-3´ |
